# Ultrasonic-ethanol pretreatment-assisted aqueous enzymatic extraction of *Akebia trifoliata* seed oil and its effects on physicochemical properties, fatty acid composition, nutritional composition, and antioxidant activity

**DOI:** 10.3389/fnut.2026.1896747

**Published:** 2026-08-12

**Authors:** Jinhua Shao, Shengmei Tang, Miaomiao Chang, Weizhen Huang, Yumiao Chen, Liyan Jiang

**Affiliations:** College of Chemistry and Bioengineering, Hunan University of Science and Engineering, Yongzhou, China

**Keywords:** *Akebia trifoliata seed oil*, aqueous enzymatic extraction, quality analysis, response surface methodology, ultrasonic–ethanol pretreatment

## Abstract

*Akebia trifoliata* seeds are regarded as a promising source for edible oil production. In this study, ultrasonic–ethanol coupled pretreatment was integrated with conventional aqueous enzymatic extraction (AEE) to improve the extraction efficiency of *Akebia trifoliata* seed oil. The highest oil yield (30.47%) was achieved under the conditions of 200 W ultrasonic power, 30 min ultrasonic treatment, and 60% ethanol-assisted AEE. Scanning electron microscopy (SEM) analysis revealed that ultrasonic–ethanol pretreatment increased the number of irregular voids in *Akebia trifoliata* seeds and facilitated the aggregation of oil droplets. Using oil yield as the response variable, the extraction parameters of AEE were further optimized through a Box–Behnken design. The optimal extraction conditions were determined as follows: enzymatic hydrolysis temperature of 51 °C, solid-to-liquid ratio of 1:3, enzyme concentration of 5.2%, and enzymatic hydrolysis time of 6.2 h. Under these optimized conditions, the oil yield reached 31.54%. Compared with oils obtained by the pressing method (PE) and Soxhlet extraction (SE), oil produced by ultrasonic–ethanol pretreatment-assisted aqueous enzymatic extraction (UEAEE) exhibited superior physicochemical properties while maintaining a similar fatty acid composition. In addition, higher levels of nutritional components and stronger antioxidant activity were observed in the oil obtained by UEAEE. The contents of total phenols, total flavonoids, and carotenoids were0.32 mg/g,149.60 mg/kg, and 52.70 mg/kg, respectively, while the DPPH free radical scavenging activity reached 80.03%. These findings indicate that UEAEE is an effective approach for improving both the yield and quality of *Akebia trifoliata* seed oil and provides valuable theoretical support for the high-value utilization of *Akebia trifoliata* seeds.

## 1 Introduction

*Akebia trifoliata* is the fruit of *Akebia trifoliata* (Thunb.) Koidz., a climbing plant belonging to the perennial and deciduous woody vine and commonly known as the “Bayuezha” (1). Its pulp is rich in nutrients, including 17 amino acids, 8 mineral elements, and a high concentration of vitamin C (26.40 mg/100 g), it has been widely used in traditional medicine for more than 2,000 years (2). In recent years, the cultivation area of *Akebia trifoliata* in China has expanded to approximately 420,000 mu (3). However, the fruit contains a large proportion of seeds, with the number of seeds per fruit ranging from 115 to 446 and the 100-seed weight varying from 8.05 to 15.59 g (4). The seed oil content ranges from 30.2 to 48.8% (5), yet most seeds are discarded as waste. Studies have shown that the unsaturated fatty acid content of *Akebia trifoliata* seed oil exceeds 70%, with oleic acid and linoleic acid as the predominant components (6). In addition, the oil exhibits excellent antioxidant stability (7), making it a promising resource for applications in the food and medicine homology sector (8).

Current methods for extracting *Akebia trifoliata* seed oil, including pressing extraction (PE), Soxhlet extraction (SE), ultrasonic-assisted extraction, and supercritical CO_2_ extraction, generally face limitations such as low extraction efficiency, safety concerns, and high capital or operating costs. Although mechanical PE is a well-established technique, its oil yield is relatively low, reaching only 16.67% (9). In contrast, SE (10) and ultrasonic-assisted extraction (11) provide higher extraction efficiencies; however, both require substantial amounts of organic solvents, which may leave solvent residues and pose risks to human health and environmental safety. Supercritical CO₂ extraction (12), while eliminating solvent residue concerns, requires considerable equipment investment and strict process control, thereby limiting its large-scale industrial application. Therefore, the development of an environmentally friendly and highly efficient extraction technology for *Akebia trifoliata* seed oil is urgently needed to improve the utilization of *Akebia trifoliata* seed resources.

Aqueous enzymatic extraction (AEE) uses water as the extraction medium, enzymes are introduced to disrupt the cell walls of oil-bearing seeds and hydrolyze complexes such as lipoproteins and lipopolysaccharides, then oil–water separation is achieved based on the differences in affinity and density between oil and water subsequently (13). AEE has attracted considerable attention because of its environmental friendliness, safety, non-toxicity, and ability to maintain product quality (14). However, despite these advantages, AEE still suffers from several limitations, particularly low oil recovery and severe emulsion formation during the extraction process (15). For example, Li et al. (16) applied AEE directly to the extraction of *Akebia trifoliata* seed oil and obtained an oil yield of only 11%, which was substantially lower than those achieved using other extraction methods. Ultrasonic-assisted extraction (UE) is considered a green and efficient technique for oil extraction with a high extraction rate, low energy consumption, and easy integration with other extraction processes (17). In recent years, ultrasound-assisted aqueous enzymatic extraction has been increasingly employed for oil recovery from various oil-bearing materials, including pomegranate seeds (18), walnuts (19), and Korean pine seeds (13). However, ultrasound can readily induce emulsification in oil–water systems, and dynamic balance between emulsification and demulsification is strongly influenced by parameters such as ultrasonic treatment time and temperature (20). Therefore, when ultrasound is applied solely during the pretreatment stage, it can alter the molecular structure of proteins, reduce the Gibbs free energy and activation energy required for subsequent enzymatic hydrolysis, and enhance enzyme–substrate interactions (21). As a result, the rate of enzymatic hydrolysis can be substantially increased. Ethanol pretreatment has also been reported to improve the permeability of plant tissues, altering cell membrane structures, reducing surface tensionand suppress emulsion formation, thereby facilitating oil extraction (22). For example, Wang et al. (23) reported that ethanol pretreatment significantly improved the demulsification efficiency of peony seed oil, resulting in a demulsification rate of up to 90.31%. Furthermore, studies have shown that combining ultrasound-assisted treatment with ethanol pretreatment and incorporating this strategy into enzymatic oil extraction can produce a synergistic effect, thereby enhancing oil recovery efficiency (20, 24).

To the best of our knowledge, no previous study has investigated the effect of ultrasound–ethanol pretreatment on AEE of oil from *Akebia trifoliata* seeds. In the present study, the effects of ultrasonic pretreatment parameters on the oil yield obtained through AEE were first systematically evaluated. Subsequently, the compositional and microstructural changes induced by the pretreatment were characterized. Response surface methodology (RSM) based on a Box–Behnken design was then employed to optimize the key extraction parameters of the AEE process. Finally, the physicochemical properties, fatty acid composition, nutritional constituents, and antioxidant activity of the oil obtained by AEE were comprehensively compared with those of oils extracted using two conventional methods, namely PE and SE.

The objective of this study was to develop an environmentally sustainable and highly efficient method for extracting oil from *Akebia trifoliata* seeds, with the aim of maximizing the retention of bioactive compounds and quality-related constituents, and to provide a robust data foundation for the further development and utilization of *Akebia trifoliata* seed oil.

## 2 Materials and methods

### 2.1 Materials

*Akebia trifoliata* seeds were supplied by Yongzhou Jingfeng Agriculture and Forestry Technology Co., Ltd. Uniform, plump, intact, and undamaged seeds were selected for the experiments. The seeds were thoroughly rinsed with clean water and air-dried before being dried to a constant weight in a constant-temperature oven at 40 °C. Subsequently, the dried seeds were crushed and passed through a 60-mesh sieve. The resulting powder was sealed and stored in a refrigerator at 4 °C for subsequent experiments.

### 2.2 Preparation of *Akebia trifoliata* seeds

#### 2.2.1 Optimization of ultrasonic–ethanol pretreatment conditions

To optimize the ultrasonic pretreatment process, the effects of ultrasonic power and treatment time on oil yield from *Akebia trifoliata* seeds were systematically investigated using a one-factor-at-a-time (OFAT) experimental design. Furthermore, based on preliminary experiments, ultrasound-ethanol pretreatment was conducted at 55 °C.

A 10.0 g sample of *Akebia trifoliata* seed powder was accurately weighed and fully immersed in 30 mL of 60% (v/v) ethanol solution (20). The suspension was then subjected to ultrasonic pretreatment using a KM-800CMP ultrasonic processor (Kunshan Meimei Ultrasonic Instrument Co., Ltd.) operating at 40 kHz and maintained at 55 °C. Systematic optimization was carried out by varying the ultrasonic power (0, 100, 200, 300, 400, and 500 W) and treatment time (10, 20, 30, 40, and 50 min). After pretreatment, the suspension was filtered, and the solid residue was collected, dried to constant weight at 40 °C, and cooled to room temperature prior to further analysis.

#### 2.2.2 Effects of different pretreatments on oil yield of *Akebia trifoliata* seeds

The oils obtained under different pretreatment conditions were classified as follows (Table 1): without ethanol addition and ultrasonic pretreatment as the unpretreated group (Ud); with ethanol addition (60% v/v)but without ultrasonic pretreatment as the ethanol pretreatment group (Et); with ultrasonic pretreatment but without ethanol addition (using water as a medium) as the ultrasonic pretreatment group (UL); and with both ultrasonic pretreatment and ethanol addition as the Ultrasonic-Ethanol pretreatment group (UL-Et).

**Table 1:** Classification and conditional parameters of various pretreatment methods for *Akebia trifoliata* seeds.

Group	Processing conditions
Ud	*Akebia trifoliata* seed powder, unprocessed
Et	Add a 60% (v/v) ethanol solution to the *Akebia trifoliata* seed powder at a solid-to-liquid ratio of 1:3 (w/v), and soak for 30 min without ultrasonication.
UL	Add distilled water to the *Akebia trifoliata* seed powder at a solid-to-liquid ratio of 1:3 (w/v), and subject the mixture to sonication for 30 min at 55 °C and 200 W.
UL-Et	Add a 60% (v/v) ethanol solution to the *Akebia trifoliata* seed powder at a solid-to-liquid ratio of 1:3 (w/v), and subject the mixture to sonication for 30 min at 55 °C and 200 W.

### 2.3 Scanning electron microscopy (SEM)

The microstructure of *Akebia trifoliata* seeds after different pretreatments was observed by a scanning electron microscope (ZEISS EVO 15, Germany). Samples were dried under vacuum and coated with a thin gold–palladium layer. Observation was carried out at 2000 × magnification under a working voltage of 20.0 kV.

### 2.4 Analysis of basic components

The basic components of untreated and ultrasonic–ethanol pretreated (*Akebia trifoliata* seed powder; 60% v/v ethanol solution; solid-to-liquid ratio of 1:3; sonication applied) were analyzed. The contents of moisture and volatile matter, ash, and protein were determined in accordance with the national standards of the People’s Republic of China, including GB/T 14489.1-2008 *Oilseeds, Determination of Moisture and Volatile Matter Content* (25), GB 5009.4-2016 *National Food Safety Standard, Determination of Ash in Foods* (26), and GB 5009.5-2025 *National Food Safety Standard, Determination of Protein in Foods* (27). In addition, the reducing sugar content was determined according to the method described by Du et al. (28), while the total sugar content was measured using the phenol–sulfuric acid method.

### 2.5 Extraction of *Akebia trifoliata* seed oil

#### 2.5.1 Ultrasonic–ethanol pretreatment-assisted AEE (UEAEE)

The UEAEE process consists of pretreatment and aqueous enzymatic extraction. After pretreatments as described above, 5.000 g of *Akebia trifoliata* seed powder was accurately weighed and mixed with ultrapure water in an Erlenmeyer flask at a predetermined solid-to-liquid ratio. The pH of the mixture was adjusted to the optimum range for enzyme activity using 0.1 mol/L HCl and 0.1 mol/L NaOH. A mixed enzyme preparation consisting of cellulase, hemicellulase, and neutral protease at a mass ratio of 1:1:1 was subsequently added (16, 29). The mixture was incubated in a constant-temperature shaker at 150 rpm at the required temperature for a specified time to facilitate enzymatic hydrolysis. Upon completion of hydrolysis, the reaction mixture was heated in a 90 °C water bath for 10 min to inactivate the enzymes. The sample was then transferred to a high-speed refrigerated centrifuge and centrifuged at 10,000 rpm for 20 min. The separated clear oil layer and emulsion layer were collected. The emulsion was frozen at −20 °C for 24 h (30), slowly thawed at room temperature for 3 h to facilitate demulsification, and then centrifuged to separate and collect the clarified oil phase. The oil yield was calculated according to Equation (1):


$$\text{Oil yield}\;(\%)=\frac{\begin{array}{l} \text{Mass of free oil}+\text{Mass of oil extracted}\;\\ \;\;\;\;\;\;\;\;\;\;\;\;\;\;\;\;\;\;\;\;\;\text{after demulsification} \end{array}}{\text{Mass of}\;\mathrm{raw}\;\text{material}}\times 100\%$$ (1)

#### 2.5.2 Soxhlet extraction (SE)

Oil extraction using a Soxhlet apparatus was performed according to the method described by Mohammadpour et al. (31), with minor modifications. Briefly, 5.0 g of *Akebia trifoliata* seed powder was accurately weighed and placed into a filter cartridge, which was then loaded into a Soxhlet extractor. Extraction was carried out with 100 mL of petroleum ether at 60 °C for 6 h. After extraction, the solvent was removed under reduced pressure using a rotary evaporator, and the crude oil was further dried in an oven at 50 °C for 4 h. The obtained oil was subsequently stored at 4 °C for further analysis.

#### 2.5.3 Pressing extraction (PE)

*Akebia trifoliata* seed oil was extracted by mechanical pressing (32). A screw press was used under conditions of 60 ± 5 °C and 50 MPa. The crude oil obtained was then centrifuged at 6,000 rpm for 10 min to remove impurities. The clarified oil was collected, packaged, and stored at 4 °C in the dark for subsequent experiments.

### 2.6 Optimization of AEE process

#### 2.6.1 Single-factor experiments

Single-factor experiments were conducted to investigate the effects of solid-to-liquid ratio, enzymatic hydrolysis time, enzymatic hydrolysis temperature, and enzyme concentration on oil yield. A 5.0 g sample of ultrasonic–ethanol pretreated *Akebia trifoliata* seed powder (Section 2.2) was accurately weighed for each experiment. Based on the optimized compound enzyme ratio and pH conditions obtained from preliminary experiments (Section 2.5.1), a mixed enzyme system consisting of cellulase, hemicellulase, and neutral protease (mass ratio 1:1:1) was added, and the extraction was carried out at pH 5. The investigated parameters were set as follows: solid-to-liquid ratio (1:1, 1:3, 1:5, 1:7, 1:9, and 1:11), enzymatic hydrolysis time (2, 4, 6, 8, and 10 h), enzymatic hydrolysis temperature (40, 45, 50, 55, and 60 °C), and enzyme concentration (1, 3, 5, 7, and 9%). Three parallel experiments were performed for each group, and the average values were calculated.

#### 2.6.2 Response surface experimental design

Oil yield of *Akebia trifoliata* seed oil was used as the response variable. Based on the single-factor experimental results, a Box–Behnken design combined with response surface methodology was employed to optimize enzymatic hydrolysis time, enzymatic hydrolysis temperature, enzyme concentration, and solid-to-liquid ratio. A quadratic regression model was established to evaluate the main effects and interaction effects of each factor.

### 2.7 Effects of different extraction methods on the quality of *Akebia trifoliata* seed oil

#### 2.7.1 Physicochemical properties

The physicochemical properties of *Akebia trifoliata* seed oil were determined in accordance with the national standards of the People’s Republic of China. Specifically, the acid value was measured according to GB 5009.229–2025, *National Food Safety Standard*: Determination of Acid Value in Foods (33); the peroxide value according to GB 5009.227–2023, National Food Safety Standard: Determination of Peroxide Value in Foods (34); the iodine value according to GB/T 5532–2022, Animal and Vegetable Fats and Oils: Determination of Iodine Value (35); and the saponification value according to GB/T 5534–2024, Animal and Vegetable Fats and Oils: Determination of Saponification Value (36). The color characteristics of the oil samples were measured using an NR10QC colorimeter (Guangdong Threenh Technology Co., Ltd.). The L* (lightness), a* (red–green coordinate), and b* (yellow–blue coordinate) values of each sample were recorded for further analysis.

#### 2.7.2 Fatty acid composition

Fatty acid composition was analyzed in accordance with GB 5009.168–2016, *National Food Safety Standard: Determination of Fatty Acids in Foods* (37).

Chromatographic conditions were as follows: a capillary FFAP column (30 m × 0.25 mm i.d. × 0.25 μm film thickness) was used. The oven temperature program was set as follows: initial temperature of 100 °C held for 2 min, increased to 180 °C at 20 °C/min and held for 2 min, then raised to 200 °C at 5 °C/min and held for 2 min, and finally increased to 230 °C at 10 °C/min and held for 20 min. The injector and detector temperatures were set at 270 °C and 280 °C, respectively. High-purity nitrogen (99.999%) was used as the carrier gas at a constant flow rate of 1.2 mL/min. Samples were injected in split mode (split ratio 20:1), with an injection volume of 1.0 μL.

#### 2.7.3 Nutritional composition

##### 2.7.3.1 Total phenols

The method was modified from Li et al. (38). Briefly, 20 mg of gallic acid standard was accurately weighed, dissolved in anhydrous ethanol, and diluted to 100 mL to obtain a 200 mg/L stock solution. Subsequently, 0, 0.2, 0.6, 1.0, 1.2, 1.6, 2.0, 2.4, and 3.0 mL of the standard solution were separately transferred into 25 mL volumetric flasks. Then, 2.5 mL of Folin–Ciocalteu reagent was added to each flask, and the mixtures were shaken thoroughly and kept in the dark for reaction. After that, 5 mL of 5% sodium carbonate solution was added, and each solution was diluted to volume with anhydrous ethanol. The mixtures were incubated at 40 °C for 30 min, and absorbance was measured at 750 nm. A calibration curve was constructed using gallic acid concentration (X) as the independent variable and absorbance (Y) as the dependent variable. The resulting regression equation was Y = 0.0257X + 0.0044, with R^2^ = 0.9903.

For sample analysis, solutions of appropriate concentration were prepared, and absorbance was determined following the same procedure as for the standard curve. The total polyphenol content was calculated by substituting the absorbance values into the regression equation, and the results were expressed as gallic acid equivalents.

##### 2.7.3.2 Total flavonoids

The method was modified from Li et al. (38). Briefly, 20 mg of rutin standard was accurately weighed, dissolved in 60% ethanol, and diluted to 100 mL to obtain a 200 mg/L stock solution. Subsequently, 0, 1, 2, 3, 4, 5, and 6 mL of the standard solution were separately transferred into 25 mL volumetric flasks. Each flask was then adjusted to 6 mL with 60% ethanol. Next, 1 mL of 5% NaNO_2_ solution was added, and the mixture was allowed to stand for 6 min. Subsequently, 1 mL of 10% Al(NO_3_)_3_ solution was added, mixed thoroughly, and left to stand for another 6 min. After that, 10 mL of 4% NaOH solution was added with thorough mixing. The solutions were then diluted to volume with 60% ethanol and allowed to stand for 15 min. Absorbance was measured at 509 nm. A calibration curve was constructed using rutin mass (X) as the independent variable and absorbance (Y) as the dependent variable. The resulting regression equation was Y = 0.4946X + 0.0201, with R^2^ = 0.9913.

For sample analysis, solutions of appropriate concentration were prepared, and absorbance was measured following the same procedure as used for the standard curve after the addition of NaNO_2_ solution. The total flavonoid content was calculated by substituting the absorbance values into the regression equation, and the results were expressed as rutin equivalents.

##### 2.7.3.3 Carotenoids

The method was modified from Szydłowska-Czerniak et al. (39). Briefly, 0.2 g of *β*-carotene standard was accurately weighed, dissolved in n-hexane, and prepared into a 20 mg/mL stock solution. The stock solution was then serially diluted to obtain a series of standard solutions (0, 0.1, 0.5, 1, 2, 5, and 10 mg/mL). Absorbance was measured at 450 nm, and a calibration curve was constructed. The resulting regression equation was Y = 4.2179X + 0.0114, with R^2^ = 0.9940.

For sample analysis, 1.0 g of oil sample was accurately weighed into a centrifuge tube, and 4 mL of n-hexane was added to ensure complete dissolution. The mixture was centrifuged at 8,000 rpm for 10 min, and the supernatant was collected. Absorbance was then measured at 450 nm using n-hexane as the blank control. The carotenoid content was calculated by substituting the absorbance values into the standard curve equation.

#### 2.7.4 DPPH free radical scavenging rate

The method was modified from Jiang et al. (40). Briefly, 1 mL of 0.1 mmol/L DPPH solution was accurately transferred into a test tube, followed by the addition of 1 mL of sample solution. The mixture was thoroughly mixed and incubated in a constant-temperature water bath at 25 °C for 60 min. Absorbance was then measured at 510 nm. For the blank control, anhydrous ethanol was used in place of the sample solution, and absorbance was determined under the same conditions.

The calculation formula is as follows:


$$\text{DPPH free radical scavenging rate}=(1-\frac{A_{1}-A_{2}}{A_{0}})\times 100\%$$

where A_1_ represents the absorbance of the reaction mixture containing the sample solution and DPPH–ethanol solution; A_2_ represents the background absorbance of the mixture of the sample solution and anhydrous ethanol; and A_0_ represents the absorbance of the blank control (DPPH solution mixed with anhydrous ethanol).

### 2.8 Data processing

All experiments were performed in triplicate, and results are presented as mean ± standard deviation (SD). Statistical significance was evaluated using one-way analysis of variance (ANOVA) at a 5% significance level (*p* < 0.05). *Post hoc* multiple comparisons were conducted using Duncan’s multiple range test. Data analysis and graphical visualization were performed using SPSS Statistics 27.0 and Origin 2024.

## 3 Results and analysis

### 3.1 Effect of ultrasonic-ethanol pretreatment on *Akebia trifoliata* seed oil

**Figure 1:**
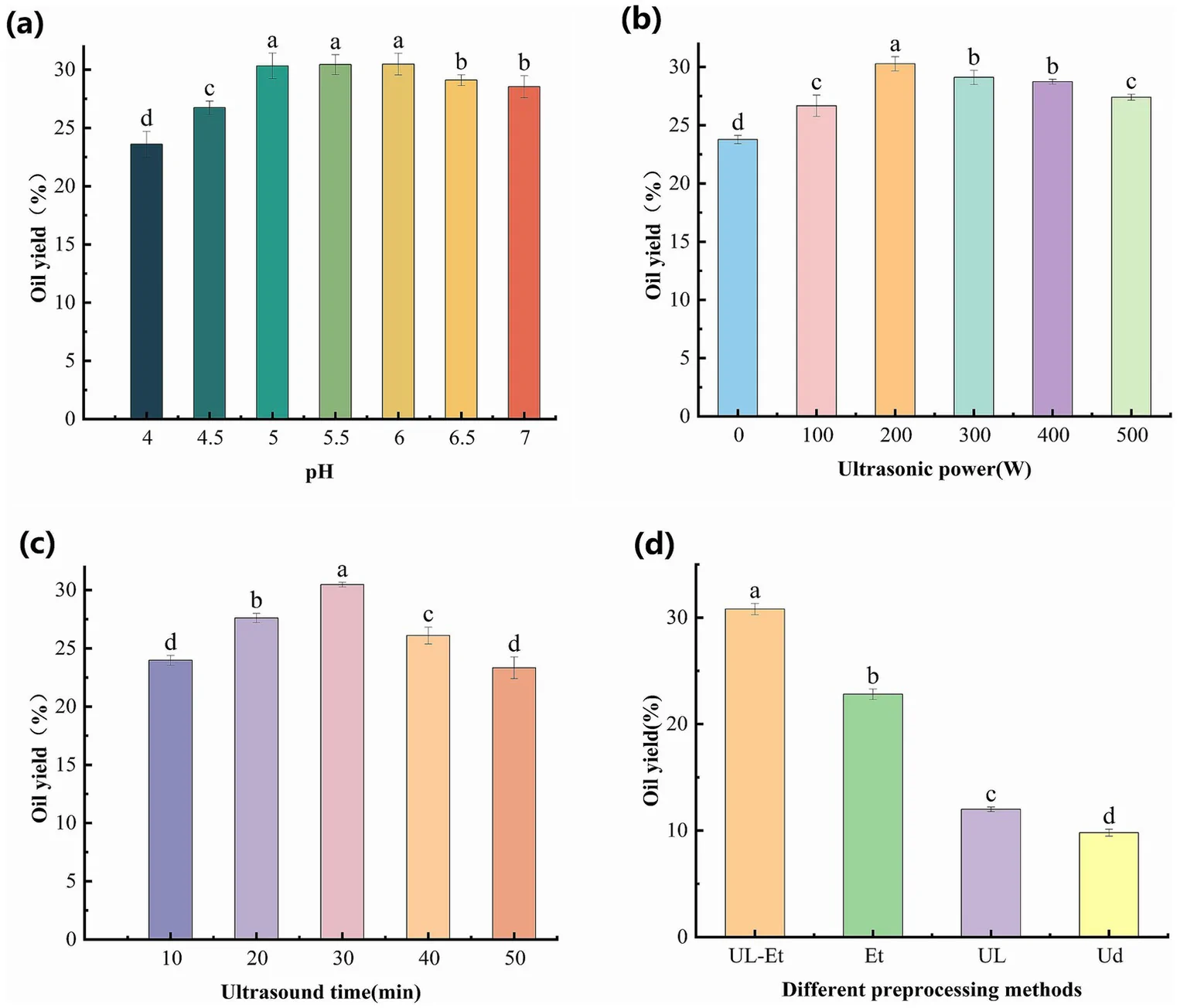
**(a)** Effect of pH on the oil yield of *Akebia trifoliata* seeds; **(b)** effect of ultrasonic power on the oil yield of *Akebia trifoliata* seeds; **(c)** effect of ultrasonic time on the oil yield of *Akebia trifoliata* seeds; **(d)** effect of different pretreatments on the oil yield of *Akebia trifoliata* seeds. Different letters in the figure indicate significant differences (*p* < 0.05).

Based on preliminary experiments, the AEE conditions after ultrasonic-ethanol pretreatment as follows: a fixed composite enzyme system consisting of cellulase, neutral protease, and hemicellulase at a mass ratio of 1:1:1; pH 5.0 (Figure 1a); a solid-to-liquid ratio of 1:3 (g/mL); a total enzyme concentration of 5% (w/w, relative to seed material); an enzymatic hydrolysis temperature of 50 °C; and a hydrolysis duration of 6 h.

Ultrasonic power is a key factor influencing extraction efficiency. As shown in Figure 1b, the oil extraction yield from *Akebia trifoliata* seeds attains its maximum when the ultrasonic power is set to 200 W. In the range of 0–200 W, increasing ultrasonic power enhanced cavitation intensity and hydrodynamic shear forces, leading to more effective disruption of cell wall structures and improved oil release (41). However, when the ultrasonic power exceeded 200 W, excessive ultrasonic power can generate an overabundance of cavitation bubbles in the solvent, reducing the effective transmission of ultrasonic energy into the medium and consequently decreasing mass transfer efficiency (42). In addition, overly intense ultrasonication promote undesirable oil degradation, including oxidation, polymerization, or isomerization, further reducing recoverable oil yield (29). Therefore, 200 W was identified as the optimal ultrasonic power for *Akebia trifoliata* seed oil extraction.

The effect of ultrasonic treatment time on oil yield is shown in Figure 1c. The oil yield increasing initially and then decreasing with prolonged ultrasonic exposure, reaching a maximum at 30 min. This behavior is consistent with the findings of Hayyan et al. (43) on argan seed oil extraction. In the early stage, extending ultrasonic time enhances cell disruption and improves solvent penetration, thereby facilitating oil release and diffusion. However, when the treatment time exceeds 30 min, prolonged ultrasonication promotes the dissolution of non-lipid components such as phospholipids, proteins, and sugars from the seed matrix, which can increase system viscosity and stabilize emulsions, thereby hindering oil separation (42). Moreover, since most intracellular oil has already been released, the diffusion gradient decreases over time, leading to reduced mass transfer efficiency (44). As a result, 30 min was determined to be the optimal ultrasonic treatment time for *Akebia trifoliata* seed oil extraction.

Figure 1d illustrates the effects of different pretreatment methods on oil yield. The overall trend followed the order UL–Et>Et>UL>Ud, with the lowest oil yield observed in the Ud group. Notably, the oil yield obtained in the Et group was higher than that in the UL group. Ultrasonic cavitation induces strong mechanical effects, which disrupts the cell wall structure of *Akebia trifoliata* seeds, this process increases membrane permeability, and facilitates the release of intracellular oil (45). In contrast, ethanol treatment contributes by dissolving lipid components within the seeds and promoting oil–water separation through an auxiliary demulsification effect, thereby creating more favorable conditions for oil release (46). Unlike ultrasonic disruption, ethanol does not rely on mechanical cell rupture; instead, ultrasound primarily induces microjet formation during cavitation collapse, which can drive water into the oil phase and promote the formation of a lotion-like emulsion. This emulsion encapsulate released oil droplets and hinder complete phase separation, thereby limiting the recoverable oil yield (47). From the perspective of the UL–Et synergistic effect, the combined application of ultrasound and ethanol pretreatment not only enhances oil release but also improves emulsion uniformity and reduces oil entrapment loss, ultimately resulting in the highest oil yield from *Akebia trifoliata* seeds. Moreover, the interaction between solvent polarity and ultrasonic cavitation facilitates mass transfer and further improves extraction efficiency (48). These results indicate that ethanol pretreatment alone significantly improves oil yield, while ultrasonic treatment provides an additional enhancement; together, their synergistic action maximizes oil recovery from *Akebia trifoliata* seeds.

### 3.2 SEM analysis

**Figure 2:**
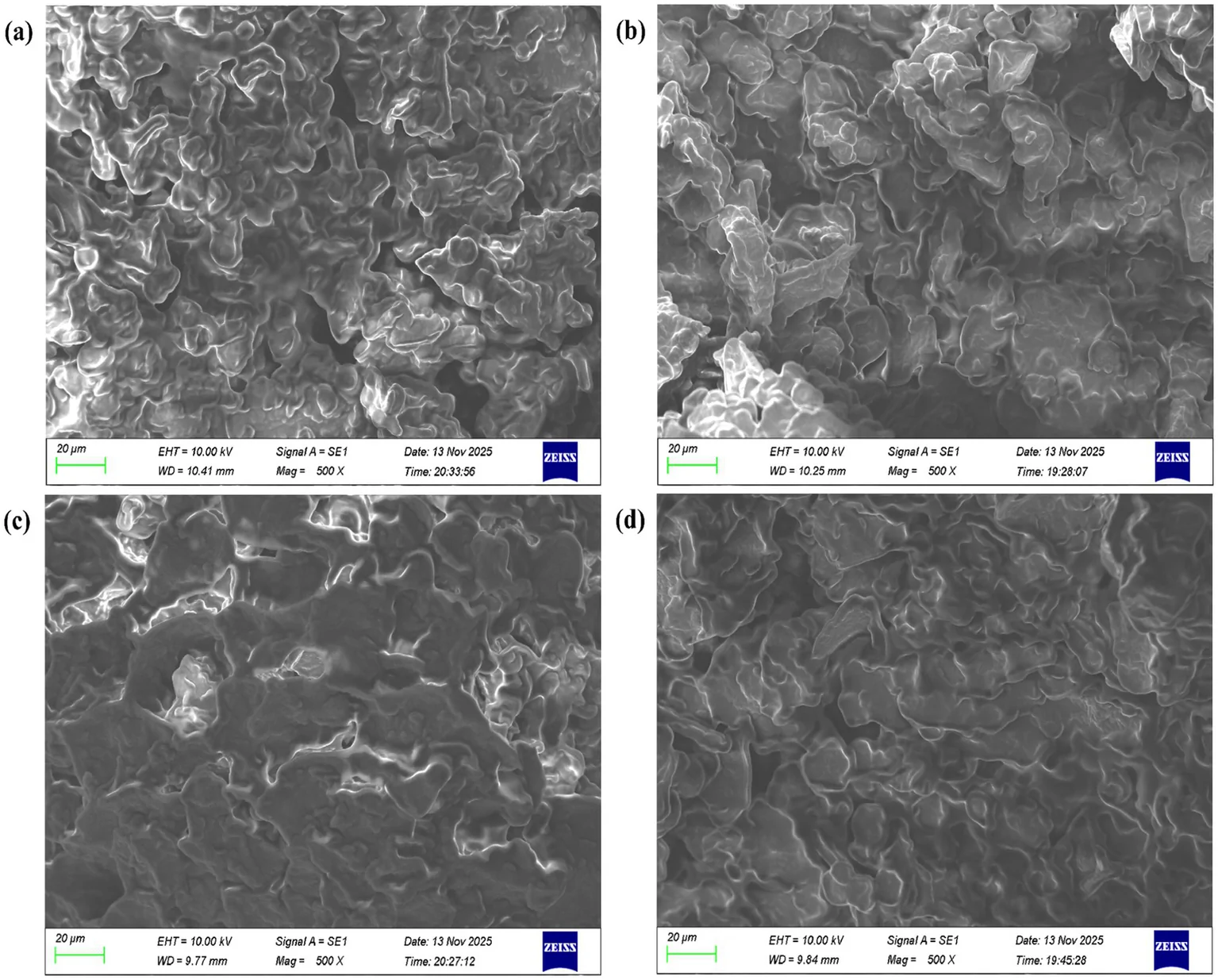
SEM analysis images of *Akebia trifoliata* seeds under different pretreatments: **(a)** Unpretreated (Ud), **(b)** Without ultrasonic pretreatment with ethanol (60%) (Et), **(c)** Ultrasonic treatment (200 W, 30 min without ethanol pretreatment) (UL), **(d)** Ultrasonic ethanol pretreatment (200 W, 30 min, 60% ethanol) (UL-Et).

The SEM images of *Akebia trifoliata* seeds subjected to different pretreatments are shown in Figure 2. As shown, the surface structure of the Ud sample (Figure 2a) is dense and intact, with tightly packed cells. This result is consistent (Figure 1d) with the observation of the lowest oil yield, suggesting that the extraction efficiency is constrained by the cell wall barrier. After Et pretreatment (Figure 2b), the pore size of the sample increased slightly, while the cellular morphology remained largely unchanged; correspondingly, the oil yield improved, suggesting that ethanol may enhance permeability to a certain extent without causing significant damage to the cell wall structure. The UL treatment (Figure 2c) induces a transition of the sample from an aggregated to a dispersed state, accompanied by an increase in pore volume and surface roughness—indicating that the mechanical action of ultrasound can effectively disrupt the intercellular aggregation structure. However, owing to its emulsification effect, the liberated oil may not be efficiently separated, thereby constraining further enhancement of oil yield. However, the UL–Et (Figure 2d) sample surface displays pronounced irregular pores and the presence of oil droplets adhering to the surface—features that correlate with its highest oil yield. The cellular architecture is severely disrupted, resulting in direct exposure of intracellular oil; this structural degradation accounts for the marked enhancement in extraction efficiency. These morphological changes are consistent with the severe cellular structural damage reported by Shao et al. (49) for perilla seeds subjected to ultrasound-assisted ethanol pretreatment.

### 3.3 Analysis of nutrient component

Compares the main components of *Akebia trifoliata* seeds before and after ultrasonic–ethanol pretreatment (Table 2). The results show that the contents of protein, reducing sugars, and total sugars decrease following pretreatment, whereas moisture and ash contents exhibit a slight increase. This observation can be attributed to the disruptive effects of ultrasound on cellular membranes, together with ethanol-induced increases in membrane permeability, which facilitate the release of small amounts of oil and protein (20). Scanning electron microscopy (SEM) analysis further supports this finding, revealing an increase in void volume in the pretreated material.

**Table 2:** Analysis and comparison of nutritional composition of *Akebia trifoliata* seeds.

Nutrient composition	Unpretreated	Ultrasound-ethanol pretreatment
Moisture (%)	3.84 ± 0.03^b^	6.40 ± 0.25^a^
Ash (%)	2.62 ± 0.13^a^	2.79 ± 0.03^a^
Protein (%)	20.40 ± 0.06^a^	18.72 ± 0.11^b^
Reducing sugar (%)	4.08 ± 0.13^a^	0.26 ± 0.01^b^
Total sugar (%)	5.32 ± 0.24^a^	1.13 ± 0.14^b^
Oil content (%)	45.19 ± 0.08^a^	40.64 ± 0.13^b^

The same letter in the same row indicates no significant difference, and different letters indicate significant difference (*p* < 0.05).

### 3.4 Optimization of AEE process

#### 3.4.1 Results of single-factor experiments

**Figure 3:**
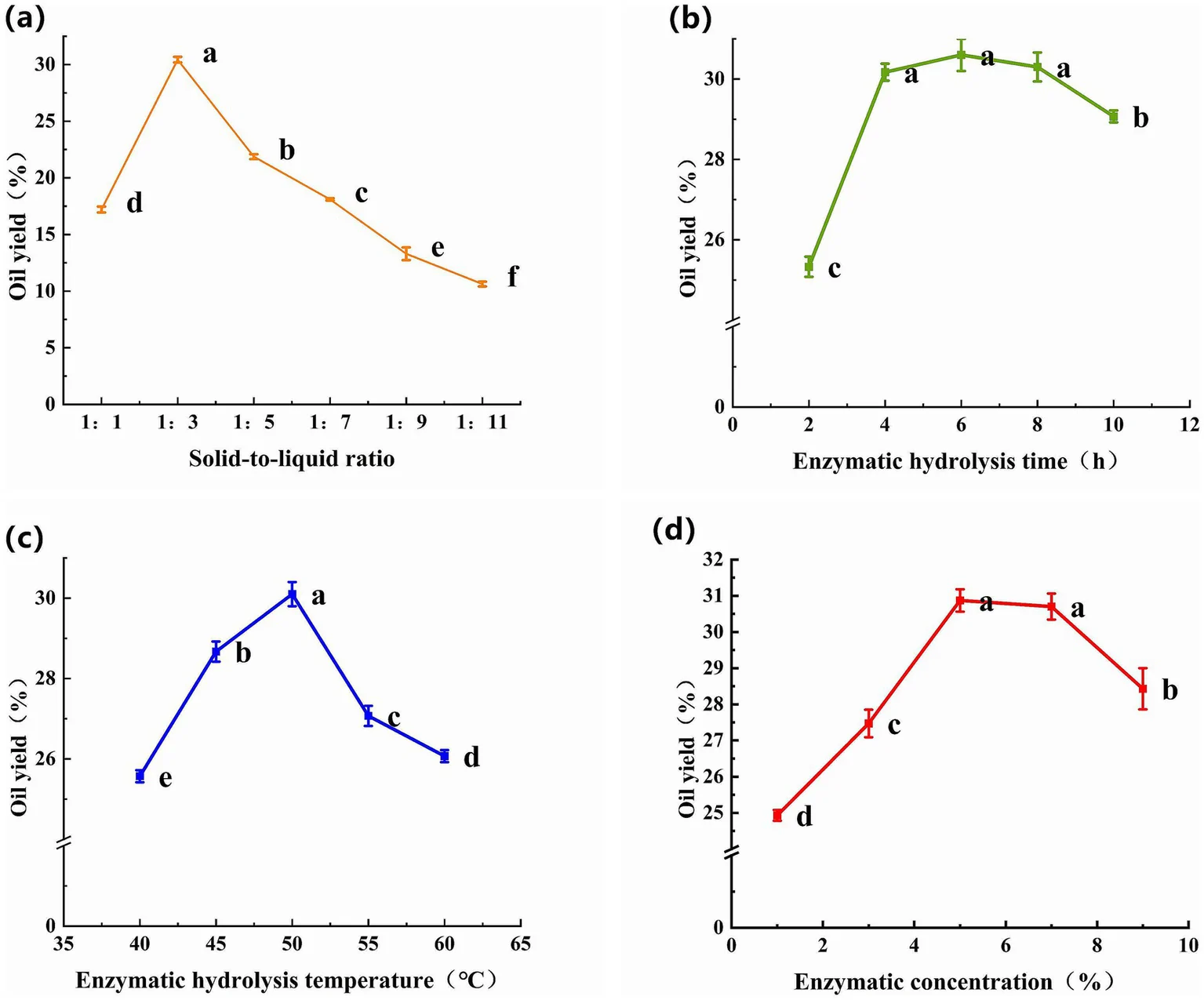
Effects of various factors on the oil yield of *Akebia trifoliata* seeds: Effect of solid-to-liquid ratio on oil yield **(a)**, effect of enzymatic hydrolysis time on oil yield **(b)**, effect of enzymatic hydrolysis temperature on oil yield **(c)**, effect of enzyme concentration on oil yield **(d)**. Different letters in the figure indicate significant differences (*p* < 0.05).

According to the previous experimental results, *Akebia trifoliata* seeds were pretreated under optimized conditions of 55 °C temperature, 200 W ultrasonic power, 30 min ultrasonic duration, and 60% ethanol concentration. Single-factor experiments were then conducted to optimize the AEE process using a fixed compound enzyme system consisting of cellulase, neutral protease, and hemicellulase at a mass ratio of 1:1:1, with the pH maintained at 5. The experimental results are presented as follows:

The effect of solid-to-liquid ratio on oil yield of *Akebia trifoliata* is shown in Figure 3a. When the solid-to-liquid ratio is below 1:3, the system viscosity is relatively high, resulting in poor mass transfer and insufficient contact between enzymes and substrates, which ultimately limits oil yield (50). When the solid-to-liquid ratio is 1:3, a moderate proportion of liquid phase facilitates enzyme penetration into the seed coat structure of *Akebia trifoliata* seeds (51). In contrast, excessively high liquid-to-solid ratios dilute both enzyme and substrate concentrations, reducing the probability of enzyme–substrate interactions and consequently decreasing hydrolysis efficiency. Moreover, higher water content can be hinder the aggregation and flotation of oil droplets, thereby complicating subsequent separation by centrifugation (19). Similarly, Díaz-Suárez et al. (52) reported that low liquid-to-solid ratios increase the viscosity of cellulase and hemicellulose treated systems, making homogenization difficult, whereas excessively high ratios reduce the effective concentrations of enzymes and substrates, thereby lowering hydrolysis efficiency and increasing downstream processing burdens. Based on these findings, a solid-to-liquid ratio of 1:3 was determined to be optimal for the extraction of *Akebia trifoliata* seed oil.

The effect of enzymatic hydrolysis time on the oil yield of *Akebia trifoliata* seeds is shown in Figure 3b. When the hydrolysis time was insufficient, the interaction between enzymes and plant cells was limited, resulting in incomplete cell wall degradation and reduced oil yield (20). Extending the hydrolysis time appropriately facilitated more complete breakdown of the cell wall structure and enhanced oil release through sustained enzymatic action, the oil yield attains maximum value (43). However, when the hydrolysis time exceeded 6 h, prolonged enzymatic reactions which decreases catalytic efficiency over time, the oil yield began to decline (53). Based on these results, 6 h was determined to be the optimal enzymatic hydrolysis time for *Akebia trifoliata* seed oil extraction.

The effect of enzymatic hydrolysis temperature on the oil yield of *Akebia trifoliata* seeds is shown in Figure 3c. Enzymes generally exhibit peak catalytic activity within a specific optimal temperature range (20). In this study, the optimal temperature ranges of cellulase, hemicellulase, and neutral protease differ, and the effective working range of the enzyme mixture is 45–55 °C, reflecting the combined effect of the three enzymes. Within this range, 50 °C represents the optimal balance point for the synergistic activity of the enzyme system. Below 50 °C, increasing temperature enhances molecular kinetic energy, promotes enzyme–substrate collisions, and accelerates the formation of enzyme–substrate complexes, thereby improving reaction efficiency and oil yield. However, when the temperature exceeds 50 °C, excessive heat induces partial denaturation and inactivation of enzymes, reduces catalytic efficiency, and disrupts system stability, ultimately resulting in decreased oil yield (54). Based on these findings, 50 °C was identified as the optimal enzymatic hydrolysis temperature for *Akebia trifoliata* seed oil extraction.

The effect of enzyme concentration on the oil yield of *Akebia trifoliata* seeds is shown in Figure 3d. At higher enzyme levels, more active sites are available to interact with the substrate and enhancing the extent of enzymatic hydrolysis (55). The results of this study indicate that the highest oil yield was achieved at an enzyme concentration of 5%. However, when the enzyme concentration exceeds the optimal range, promote sugar-related side reactions, such as caramelization (56) and increase the release of emulsifying compounds (57), these can hinder oil extraction. Therefore, an enzyme concentration of 5% was determined to be optimal for the extraction of *Akebia trifoliata* seed oil.

#### 3.4.2 Design and analysis of response surface experiments

Based on the single-factor results, enzymatic hydrolysis temperature (A), enzymatic hydrolysis time (B), solid-to-liquid ratio (C), and enzyme concentration (D) were selected as the independent variables. Ultrasonic power (200 W), ultrasonic time (30 min), and enzymatic hydrolysis pH (5) were kept constant. Oil yield was used as the response variable. A Box–Behnken design combined with response surface methodology was employed to optimize the AEE process for *Akebia trifoliata* seed oil. The experimental design matrix and corresponding results are presented in Tables 3, 4.

**Table 3:** Experimental factors and corresponding levels in the response surface methodology design.

Level	A Enzymatic hydrolysis temperature (°C)	B Benzymatic hydrolysis time (h)	C Solid-to-liquid ratio (g/mL)	D Enzyme concentration (%)
−1	45	4	1:1	3%
0	50	6	1:3	5%
1	55	8	1:5	7%

**Table 4:** Box–Behnken experimental design and corresponding results.

Number	A (°C)	B (h)	C (g/mL)	D (%)	Oil yield (%)
1	50	6	1:3	5	30.95 ± 0.23
2	50	6	1:3	5	31.53 ± 0.26
3	50	6	1:3	5	31.43 ± 0.39
4	50	6	1:3	5	31.41 ± 0.27
5	50	6	1:3	5	31.79 ± 0.14
6	45	4	1:3	5	24.20 ± 0.35
7	55	4	1:3	5	25.70 ± 0.17
8	45	8	1:3	5	24.10 ± 0.26
9	55	8	1:3	5	27.80 ± 0.26
10	50	6	1:1	3	23.50 ± 0.46
11	50	6	1:5	3	24.10 ± 0.23
12	50	6	1:1	7	25.20 ± 0.36
13	50	6	1:5	7	26.50 ± 0.26
14	45	6	1:3	3	25.35 ± 0.26
15	55	6	1:3	3	24.85 ± 0.20
16	45	6	1:3	7	24.90 ± 0.26
17	55	6	1:3	7	27.55 ± 0.17
18	50	4	1:1	5	25.00 ± 0.26
19	50	8	1:1	5	24.60 ± 0.26
20	50	4	1:5	5	24.60 ± 0.30
21	50	8	1:5	5	25.80 ± 0.26
22	45	6	1:1	5	23.90 ± 0.18
23	55	6	1:1	5	27.40 ± 0.46
24	45	6	1:5	5	26.80 ± 0.26
25	55	6	1:5	5	26.45 ± 0.22
26	50	4	1:3	3	23.40 ± 0.22
27	50	8	1:3	3	24.40 ± 0.36
28	50	4	1:3	7	24.30 ± 0.17
29	50	8	1:3	7	25.50 ± 0.13

#### 3.4.3 Response surface analysis

Response surface data were analyzed using multiple regression fitting in Design-Expert 13 software. A quadratic regression model describing the relationship between oil yield and the four independent variables (A, B, C, and D) was established as follows:

Y = 31.42 + 0.8750A + 0.4167B + 0.3875C + 0.6958D + 0.5500AB-0.9625 AC + 0.7875 AD + 0.4000 BC + 0.0500BD + 0.1750CD-2.33A^2^-3.53B^2^-2.98C^2^-3.51D^2^.

**Figure 4:**
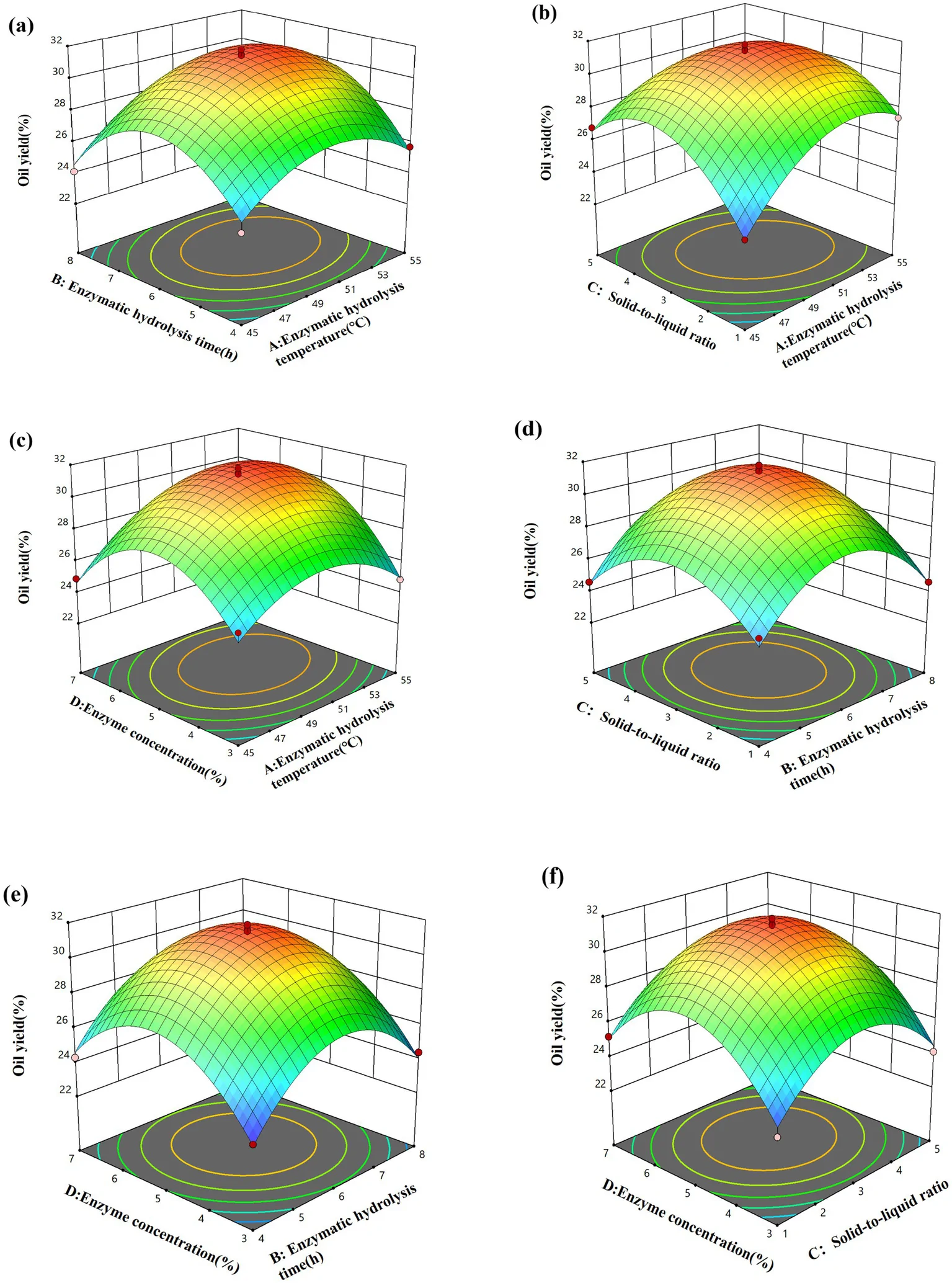
Response surface plots for the interactions among various factors. Response surface for the interaction between enzymatic hydrolysis time and enzymatic hydrolysis temperature **(a)**, solid-to-liquid ratio and enzymatic hydrolysis temperature **(b)**, enzyme concentration and enzymatic hydrolysis temperature **(c)**, solid-to-liquid ratio and enzymatic hydrolysis time **(d)**, enzyme concentration and enzymatic hydrolysis time **(e)**, enzyme concentration and solid-to-liquid ratio **(f)**.

As shown in Table 5, analysis of variance (ANOVA) indicated that the model was highly significant (*p* < 0.0001). The quadratic regression equation effectively described the relationship between the four factors and oil yield. The coefficient of determination (R^2^) was 0.9852, while the adjusted R^2^ was 0.9704, indicating a strong fit between the model and the experimental data and confirming the reliability of the results. The linear terms A and D exhibited extremely significant effects on oil yield (*p* < 0.0001 and *p* = 0.0001, respectively), while B and C showed significant effects (*p* = 0.0067 and *p* = 0.0105, respectively). In addition, all quadratic terms (A^2^, B^2^, C^2^, and D^2^) had extremely significant effects on oil yield (*p* < 0.0001). Based on the *F*-values, the influence of the factors on oil yield followed the order: enzymatic hydrolysis temperature (A)>enzyme concentration (D)>enzymatic hydrolysis time (B)>solid-to-liquid ratio (C). Significant interaction effects among factors were observed (Figure 4). The interaction between enzymatic hydrolysis temperature and solid-to-liquid ratio, as well as that between enzymatic hydrolysis temperature and enzyme concentration, was extremely significant (*p* = 0.0008 and *p* = 0.0038, respectively). The interaction between enzymatic hydrolysis temperature and enzymatic hydrolysis time was significant. In contrast, no significant interactions were observed between enzymatic hydrolysis time and solid-to-liquid ratio, enzymatic hydrolysis time and enzyme concentration, or solid-to-liquid ratio and enzyme concentration (*p* > 0.05).

**Table 5:** Response surface ANOVA results analysis.

Source of variance	Sum of squares	Degrees of freedom	Mean square	F-value	*p*-value	Significance
Model	192.59	14	13.76	66.61	< 0.0001	Significant
A-Temperature	9.19	1	9.19	44.49	< 0.0001	∗∗
B-Time	2.08	1	2.08	10.09	0.0067	∗
C-Solid-to-liquid ratio	1.8	1	1.8	8.73	0.0105	∗
D-Enzyme concentration	5.81	1	5.81	28.14	0.0001	∗∗
AB	1.21	1	1.21	5.86	0.0297	∗
AC	3.71	1	3.71	17.94	0.0008	∗∗
AD	2.48	1	2.48	12.01	0.0038	∗
BC	0.64	1	0.64	3.1	0.1002	
BD	0.01	1	0.01	0.0484	0.829	
CD	0.1225	1	0.1225	0.5932	0.454	
A^2^	35.27	1	35.27	170.79	< 0.0001	∗∗
B^2^	80.91	1	80.91	391.81	< 0.0001	∗∗
C^2^	57.43	1	57.43	278.11	< 0.0001	∗∗
D^2^	80.05	1	80.05	387.66	< 0.0001	∗∗
Residual	2.89	14	0.2065			
Lack of Fit	2.52	10	0.2521	2.72	0.1732	Not significant
Pure Error	0.3701	4	0.0925			
Cor Total	195.48	28				

∗∗The difference is highly significant (*p* < 0.01), ∗the difference is significant (*p* < 0.05).

#### 3.4.4 Verification experiment

The optimal extraction parameters predicted by the Box–Behnken model were as follows: solid-to-liquid ratio of 1:3.079 g/mL, enzymatic hydrolysis temperature of 51.047 °C, enzymatic hydrolysis time of 6.157 h, enzyme concentration of 5.247%, and a predicted oil yield of 31.581%. For practical operation, these parameters were appropriately adjusted and simplified. The final optimized extraction conditions for *Akebia trifoliata* seed oil were set as follows: solid-to-liquid ratio of 1:3 g/mL, enzymatic hydrolysis temperature of 51 °C, enzymatic hydrolysis time of 6.2 h, and enzyme concentration of 5.2%. Validation experiments showed that the oil yield reached 31.54% under the optimized conditions (Table 6), which closely matched the predicted value. This yield was also substantially higher than the 11% reported by Li et al. (16) for single AEE, demonstrating the superiority of the optimized process.

**Table 6:** Oil yield obtained in the validation experiment.

Category	1	2	3	4	5	Average value
Actual value	31.45%	31.6%	31.85%	31.1%	31.7%	31.54%

### 3.5 Effects of different extraction methods on the quality of *Akebia trifoliata* seed oil

#### 3.5.1 Physicochemical properties

Table 7 compares the physicochemical properties of *Akebia trifoliata* seed oils extracted via UEAEE, PE, and SE. The acid value (AV) and peroxide value (PV) of all three oils fall within the acceptable limits specified by the American Oil Chemists’ Society (AOCS) for edible oils. Among the three methods, SE produced oil with the highest AV and PV, likely due to prolonged exposure to elevated temperatures during extraction, which accelerates oxidative degradation (58). The iodine value (IV) is an indicator of the degree of unsaturation in oils and fats, whereas the saponification value (SV) reflects the average molecular weight (chain length) of esterified fatty acids. The UEAEE-derived oil exhibited slightly higher IV and SV than those obtained by PE and SE, suggesting better retention of unsaturated fatty acids and relatively higher extraction efficiency.

**Table 7:** Physicochemical properties of *Akebia trifoliata* seed oil extracted by different methods.

Physicochemical properties	UEAEE	PE	SE
Acid value (mg/g)	1.91 ± 0.06^a^	1.05 ± 0.01^a^	2.89 ± 0.04^a^
Peroxide value (g/100 g)	0.06 ± 0.00^b^	0.05 ± 0.00^c^	0.09 ± 0.00^a^
Iodine value (g/100 g)	117.70 ± 0.35^a^	115.22 ± 0.80^c^	114.80 ± 0.42^b^
Saponification value (mg/g)	261.51 ± 0.59^a^	259.21 ± 0.21^b^	257.27 ± 0.15^c^
L*	28.85 ± 0.88^a^	28.95 ± 0.03^a^	28.72 ± 0.09^b^
a*	0.54 ± 0.07^a^	0.89 ± 0.02^b^	1.14 ± 0.10^a^
b*	8.01 ± 0.14^a^	7.67 ± 0.10^b^	6.36 ± 0.24^c^

The same superscript letter in the same row indicates no significant difference, and different letters indicate significant difference (*p* < 0.05). L*, a* and b* represent brightness, red chroma and yellow chroma, respectively.

Color is a critical sensory attribute influencing consumer acceptance of vegetable oils. The UEAEE-extracted oil exhibits the most pronounced yellow color, a characteristic closely associated with its specific process parameters. PE oil shows the highest L* value, indicating greater brightness. SE oil presents the highest a* value, reflecting a stronger red hue, likely due to pigment dissolution under high temperature and prolonged extraction time. UEAEE oil exhibits the highest b* value, indicating a more pronounced yellow coloration, this may be due to limited chlorophyll dissolution under mild conditions, with carotenoids being the primary contributors to oil color (59).

#### 3.5.2 Fatty acid composition analysis

The data presented in Table 8 indicate that the three extraction methods have little influence on the fatty acid composition of *Akebia trifoliata* seed oil. The oil mainly consists of palmitic acid (20.87–22.24%), stearic acid (1.65–1.87%), oleic acid (42.91–44.67%), linoleic acid (28.38–30.58%), linolenic acid (0.18–0.46%), eicosenoic acid (1.23–1.62%), eicosapentaenoic acid (0.51–0.64%), and docosenoic acid (0.64–0.76%). These results are generally consistent with the fatty acid profile of *Akebia trifoliata* seed oil reported by Zhong et al. (6). Notably, relatively high levels of linolenic acid and several long-chain polyunsaturated fatty acids that have rarely been reported previously, including eicosapentaenoic acid and docosenoic acid, were detected in this study. The observed differences can be attributed to variations in raw material variety, harvest time, growth conditions, and oil extraction methods (60). They also reflect genotypic variation, as genetic mutations can modulate enzyme activity, thereby altering metabolic flux and ultimately affecting the relative proportions of fatty acids (61).

**Table 8:** Analysis of fatty acid composition of *Akebia trifoliata* seed oil extracted by UEAEE, MP, and SE.

Fatty acid	Relative content of fatty acids/%		
	UEAEE	PE	SE
Palmitic acid (16:0)	20.87 ± 0.18^c^	21.90 ± 0.18^b^	22.24 ± 0.03^a^
Stearic acid (18:0)	1.65 ± 0.01^b^	1.87 ± 0.11^a^	1.76 ± 0.07^a^
Oleic acid (18:1)	43.51 ± 0.22^b^	44.67 ± 0.32^a^	42.91 ± 0.31^c^
Linoleic acid (18:2)	30.58 ± 0.08^a^	28.38 ± 0.16^b^	30.53 ± 0.28^a^
Linolenic acid (18:3w3)	0.46 ± 0.02^a^	0.27 ± 0.07^b^	0.18 ± 0.02^c^
Eicosenoic acid (20:1)	1.62 ± 0.01^a^	1.53 ± 0.01^b^	1.23 ± 0.05^c^
Eicosapentaenoic acid (20:5)	0.56 ± 0.05^b^	0.64 ± 0.05^a^	0.51 ± 0.01^c^
Docosenoic acid (22:1)	0.76 ± 0.09^a^	0.74 ± 0.02^a^	0.64 ± 0.05^a^
Saturated fatty acid	22.52 ± 0.19^b^	23.77 ± 0.09^a^	24 ± 0.07^a^
unsaturated fatty acids	77.48 ± 0.19^a^	76.23 ± 0.09^b^	76 ± 0.07^c^

The same superscript letter in the same row indicates no significant difference, and different letters indicate significant difference (*p* < 0.05).

#### 3.5.3 Nutritional composition and antioxidant activity analysis

**Figure 5:**
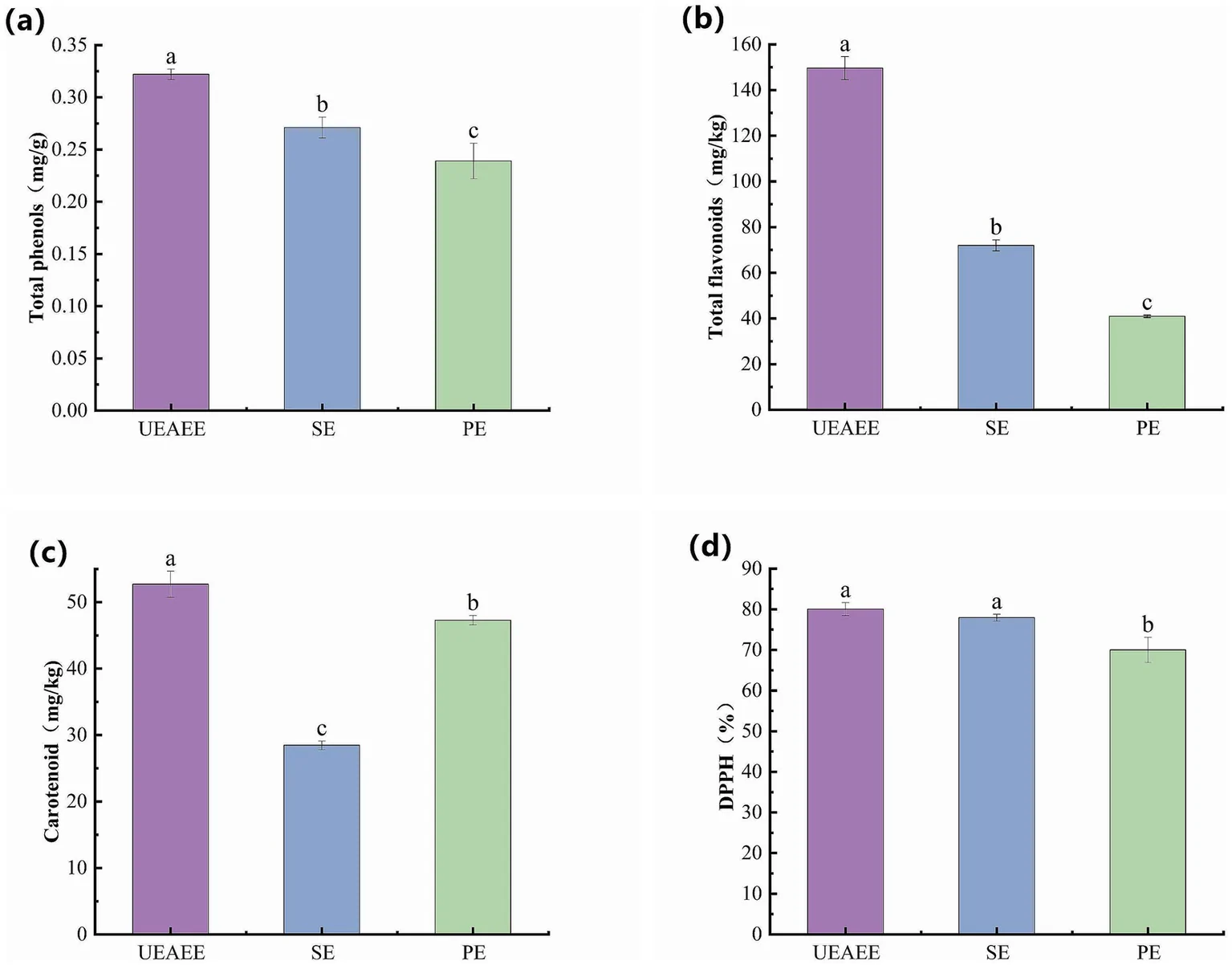
Total phenolic content **(a)**, total flavonoid content **(b)**, carotenoid content **(c)**, and DPPH radical scavenging capacity **(d)** of *Akebia trifoliata* seed oil extracted by different extraction processes. Different letters in the figure indicate significant differences (*p* < 0.05).

As shown in Figure 5a, UEAEE oil exhibits a significantly higher total phenol content than oils obtained by the other two extraction methods. This is primarily attributable to the disruptive effects of ultrasound-ethanol pretreatment on cellular integrity, coupled with enzymatic hydrolysis that cleaves the covalent linkages between phenolic compounds and cell wall polysaccharides or proteins—collectively facilitating the the yield of total phenolics (62). Although SE employs organic solvents for prolonged reflux extraction, part of the phenolic compounds undergo degradation or oxidation during heating, resulting in a lower total phenol content compared with UEAEE oil. This finding is consistent with the report of Hu et al. (29), who found that cherry seed oil extracted by AEE contained higher total phenol levels than oil obtained by SE. PE oil exhibits the lowest total phenol content, mainly because mechanical pressing lacks an effective cell wall-disruption mechanism.

As shown in Figure 5b, UEAEE exhibited the highest total flavonoid content. Ultrasonic–ethanol pretreatment promotes stronger interactions between ethanol and flavonoids, when coupled with subsequent enzymatic hydrolysis, can effectively converts bound flavonoids into free forms, significantly increasing extraction efficiency (63, 64). SE effectively extracts free flavonoids but shows limited capability in releasing bound flavonoids, whereas PE mainly relies on mechanical crushing and causes insufficient disruption of tissue structures, resulting in the lowest flavonoid release efficiency.

The carotenoid contents shown in Figure 5c follow the order UEAEE>PE>SE. Savic et al. (65) reported that heat is one of the major factors responsible for carotenoid degradation. The high extraction temperature and prolonged extraction duration involved in SE accelerate thermal degradation and cis–trans isomerization of carotenoids (66). PE induces minimal disruption to the cellular structure of seeds, thereby limiting the release of carotenoids. The UEAEE extraction system offers a stable and protective environment for carotenoids (67), while ethanol pretreatment can effectively solubilizes intracellular pigments and ultrasound-induced cavitation enhances mass transfer and extraction performance through improved solvent penetration and solute–solvent interactions (68). The synergistic action of these two mechanisms substantially improves carotenoid extraction efficiency.

The DPPH radical scavenging activity is presented in Figure 5d. UEAEE exhibited the highest DPPH radical scavenging capacity, followed by SE and PE. This finding is consistent with that of Xu et al. (69), who demonstrated that rice bran oil obtained via enzymatic extraction exhibits significantly higher DPPH radical scavenging activity than oil extracted using the Soxhlet method. This is primarily attributable to the ultrasonic ethanol pretreatment and its mild extraction conditions, which collectively enhance the extraction yield of antioxidant-active compounds and thereby improve overall antioxidant activity.

## 4 Conclusion

This study demonstrates that ultrasonic–ethanol pretreatment can effectively enhance the AEE of *Akebia trifoliata* seed oil. Compared with unpretreated samples, ultrasonic–ethanol pretreatment reduces the protein content of the raw materials and markedly disrupts the cellular structure of *Akebia trifoliata* seeds, the samples become more susceptible to enzymatic hydrolysis, while the formation of stable emulsions is reduced, thereby improving oil extraction efficiency. Based on single-factor experiments and response surface optimization, the optimal extraction conditions were determined as follows: enzymatic hydrolysis temperature of 51 °C, solid-to-liquid ratio of 1:3, enzyme concentration of 5.2%, and enzymatic hydrolysis time of 6.2 h. Under these optimized conditions, the oil yield reached 31.54%. Compared with PE and SE, UEAEE exhibited clear advantages in preserving bioactive compounds and enhancing antioxidant activity. The contents of total phenols (0.32 mg/g), total flavonoids (149.60 mg/kg), and carotenoids (52.70 mg/kg) in UEAEE oil were significantly higher than those in oils obtained by PE and SE. In addition, the DPPH free radical scavenging activity of UEAEE oil reached 80.03%, indicating markedly enhanced antioxidant capacity. Our findings demonstrate that UEAEE is a green and highly efficient oil extraction technology characterized by mild operating conditions, relatively simple equipment requirements, and the absence of residual organic solvents. However, its industrial scalability remains limited by prolonged enzymatic hydrolysis times, high costs of enzyme preparations, and constraints in ultrasonic energy transfer efficiency. To promote industrial application, future research should focus on mechanistic studies of ultrasound–enzyme interactions, optimization of integrated multi-technology processing strategies, and the development of intelligent process control systems.

## Data Availability

The original contributions presented in the study are included in the article/supplementary material, further inquiries can be directed to the corresponding author.
